# Efficacy of Low‐Dose Intravenous Ketamine With Spinal Anesthesia for Postoperative Pain Control in Total Knee Arthroplasty: A Randomized Clinical Trial

**DOI:** 10.1155/prm/8176907

**Published:** 2026-06-17

**Authors:** Arash Sherafat Vaziri, Sina Javidmehr, Alireza Arvin, Arash Heroabadi, Fardis Vosoughi, Sohrab Keyhani, Maryam Salimi, Mir Saeed Yekaninejad, Hassan Zolghadr, Seyedeh Reihaneh Hosseini

**Affiliations:** ^1^ Center for Orthopedic Trans-Disciplinary Applied Research, Tehran University of Medical Sciences, Tehran, Iran, tums.ac.ir; ^2^ Department of Orthopaedic Surgery, Sina Hospital, Tehran University of Medical Sciences, Tehran, Iran, tums.ac.ir; ^3^ Brain and Spinal Cord Injury Research Center, Tehran University of Medical Sciences, Tehran, Iran, tums.ac.ir; ^4^ Department of Orthopaedics and Trauma Surgery, Shariati Hospital, Tehran University of Medical Sciences, Tehran, Iran, tums.ac.ir; ^5^ Akhtar Orthopedic Hospital, Shahid Beheshti University of Medical Sciences, Tehran, Iran, sbmu.ac.ir; ^6^ Department of Epidemiology and Biostatistics, School of Public Health, Tehran University of Medical Sciences, Tehran, Iran, tums.ac.ir

**Keywords:** ketamine, postoperative pain, spinal anesthesia, total knee arthroplasty

## Abstract

**Background:**

The efficacy of low‐dose intravenous (IV) ketamine as an adjunct analgesic to spinal anesthesia in total knee arthroplasty (TKA) remains uncertain. This study aims to assess ketamine’s impact on postoperative pain level and opioid consumption.

**Methods:**

In a randomized, triple‐blind, placebo‐controlled trial conducted at Shariati Hospital, Tehran, Iran, 65 patients undergoing unilateral TKA were assigned to receive either 0.5 mg/kg IV ketamine or saline. The primary outcome was cumulative opioid consumption in the first 24 h. Secondary outcomes were visual analog scale (VAS) pain scores at 2, 6, 12, and 24 h and at 2 and 6 weeks, knee range of motion at discharge, operative time, length of stay, and adverse effects.

**Results:**

Median 0‐ to 24‐h opioid use was similar (ketamine 10 [range 10–17.5] vs. placebo 10 [range 10–15] morphine milligram equivalents [MME]; *p* = 1.00). VAS scores were numerically lower with ketamine at all time points, but no comparison remained significant after adjustment for multiple comparisons, and there was no time × treatment interaction. No significant differences were observed in other secondary outcomes, including knee flexion and extension, operative time, and length of stay. Side effects were minimal and comparable between groups.

**Conclusions:**

Low‐dose IV ketamine shows no measurable analgesic benefits in the long term, and it does not significantly reduce pain or opioid use after TKA within a multimodal pain protocol. Further research is needed to explore various analgesia strategies and follow‐up durations to optimize pain management.

**Trial Registration:** Iranian Registry of Clinical Trials: IRCT20220315054298N1

## 1. Introduction

With an aging population and increasing obesity rates, joint replacement has become a common procedure to relieve pain and improve quality of life in patients with severe joint diseases such as osteoarthritis (OA) [1, 2]. Postoperative pain remains a major challenge, particularly after total knee arthroplasty (TKA), one of the most painful orthopedic procedures [3]. Despite advances in pain management, over 80% of TKA patients still experience inadequate pain control, which can impair early mobilization, delay recovery, prolong hospitalization, and increase the risk of chronic pain and prolonged opioid use [4–7].

Multimodal analgesia is therefore widely used, with opioids remaining a central component due to their potent analgesic effects [8]. However, opioids are associated with adverse effects such as constipation, nausea, respiratory depression, addiction, and tolerance with prolonged use [9]. Additionally, prolonged opioid use may contribute to opioid‐induced hyperalgesia through enhanced NMDA receptor (NMDAR) activation in the spinal dorsal horn [10].

Ketamine, a noncompetitive NMDAR antagonist, has been proposed as an adjunct analgesic because of its ability to reduce central sensitization and opioid requirements at low doses [11–13]. Its analgesic efficacy, however, appears to depend on factors such as dose, route of administration, and background anesthetic technique [14–17].

This randomized controlled trial (RCT) study aimed to assess the efficacy and safety of low‐dose intravenous (IV) ketamine, administered alongside spinal anesthesia, for pain reduction following TKA. We hypothesized that low‐dose IV ketamine, in combination with spinal anesthesia, would effectively reduce pain and opioid consumption in TKA patients, without increasing adverse effects.

## 2. Methods

### 2.1. Study Design

Between June and August 2022, a randomized, triple‐blind, placebo‐controlled clinical trial was conducted at Shariati Hospital, Tehran University of Medical Sciences, Tehran, Iran. The study aimed to evaluate the efficacy and safety of IV ketamine (adjunct to spinal anesthesia) in managing postoperative pain following TKA. The trial adhered to the Declaration of Helsinki, the study protocol was approved by the Institutional Review Board of Tehran University of Medical Sciences (IR.TUMS.MEDICINE.REC.1400.1408), and the article adheres to the applicable CONSORT guidelines (Completed CONSORT Checklist is available as supporting information). Written informed consent was obtained from all participants before inclusion. The study was registered prior to patient enrollment at the Iranian Clinical Trials Registry (Principal investigator: Sina Javidmehr, Date of registration: 27th of April 2022). All study data were recorded on standardized case report forms (CRFs), and all investigators, care providers, and data collectors were blinded to group assignments (triple‐blind design).

### 2.2. Participants

Participants were male and female inpatients, aged 18 to 85, undergoing primary unilateral TKA for degenerative end‐stage OA of the knee. Eligibility requires a score of > 2 on the Kellgren–Lawrence scale, a common method for classifying the OA severity into five grades [18]. The following were the exclusion criteria of the study: (1) patients classified as American Society of Anesthesiologists (ASA) IV or V; (2) body mass index greater than 40 kg/m^2^ (morbid obesity, excluded due to higher perioperative risk and potential confounding in pain outcomes [19]); (3) rheumatoid arthritis; (4) allergy or contraindication to opioid analgesics or amide/sulfa anesthetics; (5) inability to understand the visual analog scale (VAS); (6) prior ketamine usage or prolonged opioid use (> 10 mg morphine milligram equivalents [MMEs] per day); (7) previous history of knee arthroplasty, prior surgery on the ipsilateral knee within 6 months or simultaneous bilateral arthroplasty; and (8) patients undergoing general anesthesia or a nerve block. Additionally, (9) patients with an ejection fraction < 30%; (10) creatinine clearance < 30 mL/min; (11) chronic liver disease; (12) neurological or psychiatric disorders (e.g., bipolar disorder, post‐traumatic stress disorder, schizophrenia); and (13) chronic alcohol abuse were excluded due to ketamine contraindications. These criteria were assessed by the research team (including an anesthesiologist and a senior orthopedic surgeon who performed the TKA surgery) during the preoperative clinic visit using patient history and physical examination.

### 2.3. Surgical Technique

Spinal anesthesia was performed by an experienced anesthesiologist using an intrathecal injection of 15 mg isobaric bupivacaine 0.5% combined with 5 mcg sufentanil and 2 mg IV midazolam. Intraoperative sedation was maintained with propofol (50 mcg/kg/min). The senior surgeon performed cemented primary TKA using Persona The Personalized Knee components (Zimmer Biomet), without patient‐specific instruments or robotic assistance. The patella was not resurfaced.

### 2.4. Randomization and Blinding

Participants were randomized in a 1:1 ratio using block randomization with a block size of four, generated via a digital random number generator (https://www.randomization.org). An independent team member not involved in the study handled the randomization process. The allocation was concealed using opaque, sealed, sequentially numbered envelopes containing instructions for syringe preparation. An anesthesiology technician prepared identical 5‐mL syringes, ensuring the solutions were indistinguishable in appearance and odor. This technician was not involved in any further patient care or assessments. All patients, care providers, and outcome assessors remained blind to the treatment assignments throughout the study (triple‐blind).

### 2.5. Interventions (Study Medication)

Eligible patients were randomized into two groups. Group A (experimental) received 0.5 mg/kg of IV ketamine (PANPHARMA GmbH, Germany), diluted to 5 mL with normal saline, administered over 2 min immediately after confirmation of the spinal block. Group B (placebo) received 5 mL of 0.9% IV saline. Intraoperative pain and hemorrhage control included two local injections. The first consisted of 1 g tranexamic acid, 30 mg ketorolac, and 60 mg bupivacaine, injected into periarticular tissues and the posterior capsule. The second injection, administered after arthrotomy closure, contained 2 g of tranexamic acid and 40 mg of bupivacaine.

Postoperative pain management included 30 mg IV ketorolac and 5 mg IM methadone in the recovery room. Patients with psychotomimetic effects, such as hallucinations or delusions, received 1 mg IV midazolam. For ongoing postoperative analgesia, patients were administered 5 mg IM methadone every 12 h, 30 mg IV ketorolac every 8 h, and 325 mg oral acetaminophen every 6 h for the first 24 h. Rescue doses of 50 mcg IV fentanyl were provided if the VAS score exceeded five, with repeat dosing every 10 min, if necessary. Postoperative opioid use was recorded as daily MME following CDC guidelines, using the conversion factors provided in Supporting Appendix S1.

### 2.6. Outcomes

#### 2.6.1. Primary Outcome

The primary outcome was cumulative opioid consumption during the first 24 postoperative hours, expressed as MME. MME values were calculated in milligrams (mg) using standardized conversion factors for oral morphine equivalency. All opioids administered, whether intravenously or orally, were converted to this single oral morphine equivalent to allow for aggregate analysis.

#### 2.6.2. Secondary Outcomes

The main secondary outcome of the study was subjective postoperative pain perception, measured using the VAS (on a scale from 0 [*no pain*] to 10 [*most extreme pain*]). Pain was assessed at seven time points: preoperatively (baseline), at 2, 6, 12, and 24 h postoperatively during hospitalization, and again at 2 and 6 weeks postoperatively during follow‐up visits at the orthopedic clinic. All VAS score measurements were taken with the patient at rest (not during movement or physiotherapy) to ensure a standardized assessment environment and to account for clinical mobility restrictions in the early postoperative phase. Assessments were performed by a blinded assessor (a nurse or orthopedic resident unaware of group allocation) to ensure consistency in pain assessment.

Other secondary outcomes included knee flexion and extension range, length of hospital stay (in days), surgery time (in minutes), and adverse effects of the drug, such as headache, dizziness, nausea, vomiting, itching, agitation, depression, delirium, hallucination, and amnesia. The secondary outcomes were documented at discharge.

### 2.7. Sample Size

A power analysis, based on prior data [20, 21] on ketamine’s opioid‐sparing effect, indicated that a minimum of 44 participants (22 per group) was required to detect a 40% reduction [22] in 24‐h morphine consumption between the groups, with 90% power and a significance level of 0.05. To account for possible attrition in this longitudinal study, the sample size was increased by 30%, resulting in a minimum of 60 participants. To randomize already consented/scheduled patients, recruitment continued to 65 participants and then closed, with no interim analyses or unblinding, and all activities were conducted within preapproved resources.

### 2.8. Statistical Analysis

Data were analyzed using SPSS Version 21 (Chicago, USA). Data normality was assessed via the Shapiro–Wilk test. Continuous variables were expressed as mean ± standard deviation (SD) for normally distributed data, or as median and interquartile range (IQR) for nonnormal distributions. Categorical variables were reported as frequencies and percentages. To compare continuous variables between groups, independent *t* tests were employed for normally distributed variables, while the Kolmogorov–Smirnov test was used for nonnormally distributed variables. At the same time, chi‐square or Fisher’s exact tests were applied for categorical variables. Moreover, between‐group comparisons for VAS scores at each postoperative time point (2, 6, 12, 24 h; 2 and 6 weeks) were adjusted with the Holm–Bonferroni method. For the longitudinal analysis, a repeated‐measures general linear model assessed VAS trajectories, testing main effects of time and treatment and their interaction; when Mauchly’s test indicated a violation of sphericity, Greenhouse–Geisser corrections were applied. Given the borderline imbalance in baseline age between groups, a prespecified sensitivity analysis was performed using linear regression to evaluate the association between age and cumulative opioid consumption in the first 24 postoperative hours. Statistical significance was set at *p* < 0.05.

## 3. Results

### 3.1. The Sociodemographic and Baseline Characteristics of Participants

**FIGURE 1:**
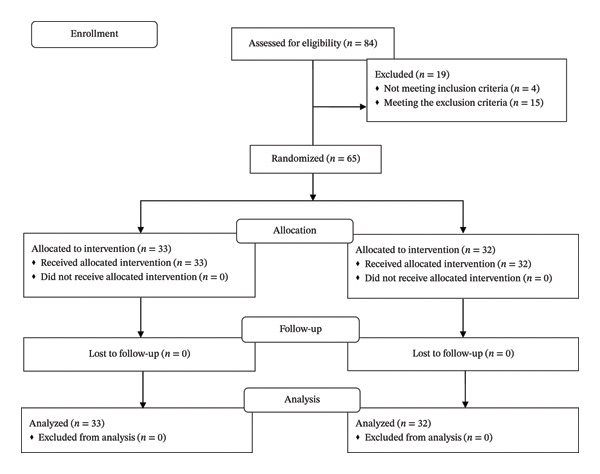
Consort flow diagram of patients’ enrollment, allocation, follow‐up, and analysis.

A total of 84 patients were screened, of whom 19 were excluded before randomization: four did not meet inclusion criteria, and 15 met exclusion criteria related to comorbidities and prior treatments. Sixty‐five patients were randomized: 33 to the IV ketamine group and 32 to the IV saline (placebo) group. All participants completed the trial, with no discontinuations in either group (Figure 1).

Randomization produced comparable baseline profiles in the ketamine and placebo groups, including age, sex distribution, BMI, and preoperative VAS score (Table 1).

**TABLE 1:** Baseline characteristics of the patients in the intervention and placebo groups.

Characteristic	Ketamine group (*N* = 33)	Placebo group (*N* = 32)
Age (years), mean ± SD	63.6 ± 8.6	67.6 ± 7.7
BMI (kg/m^2^) mean ± SD	29.0 ± 4.0	28.4 ± 5.8
Gender (*n*, %)		
Male	8 (24.2%)	6 (18.8%)
Female	25 (75.8%)	26 (81.2%)
Preoperative VAS, mean ± SD	8.2 ± 1.2	8.5 ± 1.2

Abbreviations: BMI, body mass index; SD, standard deviation; VAS, visual analog scale.

### 3.2. Primary Outcome

The prespecified primary endpoint was cumulative opioid consumption in the first 24 h, expressed as MME. Median 0–24 h MME was 10 mg (range 10–17.5, IQR 0) in the ketamine group and 10 mg (range 10–15, IQR 0) in the placebo group. The unadjusted between‐group difference was not significant (*p* = 1.00) (Table 2). Given the borderline imbalance in baseline age between groups, sensitivity analysis indicated that age was not significantly associated with cumulative opioid use (*B* = −0.016, standardized *β* = −0.067, *p* = 0.60; 95% CI: −0.077–0.044). Adjustment for age did not alter the estimated between‐group difference in opioid consumption, and the primary outcome remained unchanged.

**TABLE 2:** Comparison of primary and secondary outcomes between the ketamine and placebo groups.

Outcome	Ketamine group (*N* = 33)	Placebo group (*N* = 32)	*p* value
Cumulative morphine consumption 24 h (mg oral MME), median [range, IQR]	10 [10–17.5, 0]	10 [10–15, 0]	NS
Knee flexion at discharge (degrees), median [range, IQR]	100 [0–120, 18]	95 [80–120, 10]	NS
Knee extension at discharge (degrees), median [range, IQR]	0 [0–90, 5]	0 [0–10, 5]	NS
Length of stay (days), median [range, IQR]	3 [2–4, 1]	2 [2–5, 1]	NS
Surgery time, mean ± SD	117.9 ± 22.4	119.2 ± 24.2	NS

*Note:* Continuous variables with normal distribution are presented as mean ± SD, while those with nonnormal distribution are presented as median [range, IQR]. *p* values are obtained through the Kolmogorov–Smirnov test for continuous variables with nonnormal distribution and independent samples *t* test for those with normal distribution.

Abbreviations: IQR, interquartile range; MME, morphine milligram equivalents; NS, not significant; ROM, range of motion; SD, standard deviation.

### 3.3. Secondary Outcomes

#### 3.3.1. Pain Outcomes

**FIGURE 2:**
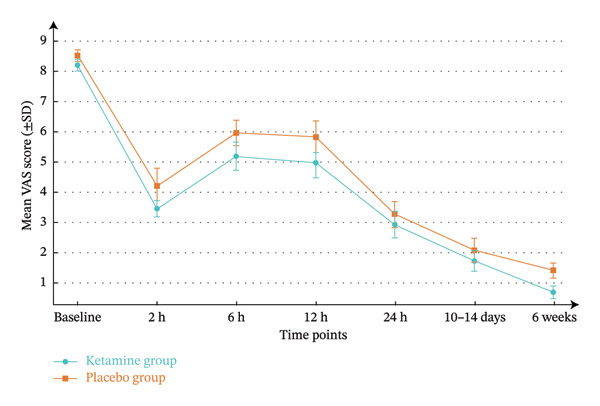
Mean visual analog scale (VAS) pain score over time for the ketamine vs. placebo groups.

At the six postoperative time points (2, 6, 12, and 24 h; 2 and 6 weeks), mean VAS scores were numerically lower in the ketamine group, but between‐group differences were small. After Holm–Bonferroni adjustment across the six comparisons, no time‐specific difference remained significant. The repeated‐measures analysis (Greenhouse–Geisser correction) also indicated no significant time × treatment interaction on VAS scores (*F* = 0.22, df = 4.38, *p* = 0.93). Figure 2 illustrates the pain trajectories; numeric labels adjacent to each point show the mean ± SD for each group at each time point.

#### 3.3.2. Other Secondary Outcomes

Postoperative knee flexion and extension demonstrated no significant differences between groups (flexion: *p* = 0.23; extension: *p* = 1.00). Surgery duration was also similar between groups (*p* = 0.81). The between‐group difference for length of stay was not statistically significant either (*p* = 0.12) (Table 2). Among the reported side effects, only four participants experienced nausea, with two in each group, showing no significant difference.

## 4. Discussion

Cumulative opioid consumption during the first 24 postoperative hours did not differ between the ketamine and placebo groups. Although the mean VAS pain scores were consistently lower in the ketamine group across all time points, these differences were small and not statistically significant. Additionally, other secondary outcomes, including knee flexion, extension, and length of stay, were comparable between groups, indicating no meaningful improvement in early functional recovery. Adverse events were minimal and similar in both groups. Overall, within a spinal anesthesia‐based multimodal analgesic regimen, low‐dose IV ketamine was well tolerated but did not confer clinically or statistically significant benefits in postoperative pain control or opioid consumption following TKA.

As the global demand for TKA continues to rise, the need for effective postoperative pain management, one of the most challenging aspects of orthopedic surgeries, becomes increasingly critical [23]. This study underscores the limited role of ketamine in multimodal pain management for TKA. Ketamine blocks NMDARs, reducing central sensitization by inhibiting pain transmission to the dorsal horn. Spinal anesthesia, however, blocks afferent nerve impulses at the peripheral level, decreasing excitatory neurotransmitters like glutamate and reducing NMDAR activity [24]. This makes ketamine’s additional effect less significant, as its target receptors are already minimally active.

Furthermore, spinal anesthesia causes vasodilation and blood pooling in the lower extremities, potentially affecting ketamine’s distribution, despite its rapid absorption [25]. These factors are likely to contribute to the reduced efficacy of the ketamine in postoperative pain control. Interestingly, a narrative review stated that when patients received basic multimodal analgesia (NSAID, paracetamol, and/or regional analgesia), systemic ketamine failed to produce a significant additional reduction in pain [26].

In evaluating the efficacy of ketamine under spinal anesthesia, a 2012 RCT in Brazil involving 56 patients undergoing TKA compared IA S‐(+)‐ketamine at doses of 0.25 mg/kg and 0.5 mg/kg to saline. While the lower dose led to reduced pain scores and a decreased need for rescue analgesia, there was no statistically significant difference in overall pain relief between the groups [15]. Similarly, a 2019 clinical trial in the United States with 91 patients showed that intraoperative infusion of ketamine at subanesthetic doses provided no significant short‐ or long‐term benefits for pain relief or opioid consumption following TKA. Although some pain reduction was noted, there were no differences in maximum pain scores or overall opioid use between the ketamine and placebo groups. These findings imply that ketamine, particularly in combination with spinal anesthesia, may not confer a meaningful advantage in postoperative pain management after TKA [16].

Conversely, our findings contrast with several prior studies reporting opioid‐sparing or analgesic effects of ketamine [14, 27–29]. These discrepancies appear to be primarily explained by differences in dose, route of administration, timing, and background anesthesia, all of which critically influence ketamine’s pharmacodynamic impact.

Many studies reporting positive analgesic or opioid‐sparing effects of ketamine in TKA were conducted under general anesthesia, where nociceptive transmission and central sensitization remain active throughout surgery [14, 27, 28]. Under these conditions, ketamine’s antagonism of NMDARs may more effectively modulate perioperative excitatory signaling and reduce postoperative pain and opioid requirements. Notably, the majority of these studies conducted under general anesthesia also employed continuous ketamine infusions, which may further confound the independent contribution of background anesthetic technique.

Some studies demonstrating positive effects employed continuous infusions or higher cumulative doses of ketamine rather than a single bolus. For instance, a 2005 RCT in France with 40 patients revealed that a small dose of bolus IV ketamine followed by continuous infusion of ketamine intra‐ and postoperatively significantly decreased morphine consumption (45 vs. 69 mg, *p* < 0.02) and accelerated knee flexion recovery (90° achieved in 7 vs. 12 days, *p* < 0.03) post‐TKA [27]. Similarly, a 2009 French RCT involving 75 patients compared bolus intraoperative injection followed by continuous IV infusion of nefopam and ketamine until the second day postoperative for pain control, showing that both reduced morphine consumption and pain scores, but ketamine was more effective in facilitating improved knee flexion by Postoperative Day 3 [28]. In contrast, bolus‐only regimens, particularly at low doses, may provide insufficient exposure to sustain NMDAR antagonism beyond the immediate intraoperative period. Our protocol, intentionally designed to minimize adverse effects, may therefore have limited ketamine’s capacity to influence postoperative nociceptive processing meaningfully.

Positive studies have also utilized regional or local routes, including intra‐articular (IA) ketamine, which may produce higher local concentrations at relevant nociceptive sites. A Chinese clinical trial in 2018 with 84 TKA patients compared IA ketamine, bupivacaine, and their combination to saline, finding that the ketamine plus bupivacaine group had significantly lower pain scores, reduced opioid consumption, shorter ambulation times, and improved knee flexion [29]. In contrast, systemic IV ketamine may exert more diffuse and transient effects, particularly when administered as a single bolus.

The type of ketamine administered may also influence analgesic outcomes. A 2001 RCT in Germany involving 37 TKA patients found that epidural S‐(+)‐ketamine combined with ropivacaine significantly reduced VAS pain scores and decreased ropivacaine consumption at 24 and 48 h postoperatively, with no significant differences in adverse events. Additionally, patients rated the quality of pain therapy higher with this combination [30]. S‐(+)‐Ketamine, the pharmacologically more active enantiomer of racemic ketamine, exhibits higher affinity for NMDARs and greater analgesic potency at lower doses, while being associated with fewer psychomimetic side effects [13]. In contrast, many studies, including the present trial, have employed racemic ketamine administered systemically, which may result in reduced analgesic efficacy.

Several systematic reviews and meta‐analyses have examined the efficacy of ketamine in postoperative pain management for orthopedic surgeries, yielding mixed results. A 2015 systematic review evaluated the effects of perioperative and intraoperative ketamine on chronic postoperative pain after major surgeries. In line with our findings, the analysis of 10 studies, including two focusing on orthopedic procedures, found insufficient evidence to support a significant reduction in chronic pain due to perioperative ketamine administration. The only notable result was a marginally significant reduction in postoperative pain at rest after 1 month [11]. Moreover, a 2015 US review article of 39 clinical trials assessing the use of low‐dose IV ketamine for postoperative analgesia found that ketamine reduces opioid consumption by 40%. While ketamine consistently demonstrated opioid‐sparing effects, its impact on pain scores was less conclusive [31].

Conversely, a 2020 meta‐analysis of multiple studies demonstrated that patients receiving perioperative ketamine during TKA/THA had significantly lower VAS scores at 6, 12, 24, and 48 h postoperatively. Additionally, there was a reduction in morphine consumption at 24 and 48 h after surgery, confirming ketamine’s efficacy and safety as an adjunct analgesic [32]. Another 2019 meta‐analysis of six RCTs confirmed that ketamine significantly reduced early postoperative pain and opioid use after TKA, with fewer gastrointestinal complications and no increased risk of thrombosis, supporting ketamine’s safety and efficacy in multimodal pain management [20]. Finally, a 2023 meta‐analysis focusing on intravenously administered ketamine confirmed its effectiveness in reducing opioid consumption in both TKA and THA, and, in four of seven studies, decreasing postoperative pain [33].

### 4.1. Limitations and Future Directions

Although 65 patients were included, the study was powered to detect a relatively large opioid‐sparing effect (40%) based on prior literature [31]. The between‐group difference in opioid consumption observed in the present study was substantially smaller than this assumed effect size, raising the possibility of insufficient statistical power to detect modest but potentially clinically relevant differences and an increased risk of Type II error. Accordingly, the absence of statistically significant differences should be interpreted with caution. Furthermore, relying solely on VAS scores for pain assessment might not capture the full multidimensional experience of pain, which encompasses sensory, emotional, and cognitive components. Additionally, the follow‐up period primarily focused on immediate postoperative opioid use and pain control, which may not capture the long‐term effects of ketamine on chronic pain development. The concurrent use of other pain management strategies could act as potential confounding factors, making it challenging to discern the isolated effects of ketamine. Such effective background analgesia plausibly reduced between‐group separability on opioid consumption, even if any opioid‐sparing effect was present. However, it would be unethical to withhold analgesics from patients. Moreover, we did not have detailed data on preoperative opioid use (although we excluded high‐dose chronic opioid users [> 10 MME/day]), so we could not tell whether opioid‐naive and opioid‐tolerant patients responded differently to ketamine. Finally, conducting the trial at a single institution may limit the diversity of the patient population and the external validity of the results.

Given the potential variability in ketamine’s efficacy across administration routes, individual patient factors, and the anesthesia type, future research should focus on large‐scale, multicenter, randomized controlled trials directly comparing the IA and IV routes of ketamine administration. These studies should also compare the use of general versus spinal anesthesia. Such research would provide valuable insights into the optimal conditions for ketamine’s use in postoperative pain management, allowing for more tailored and effective multimodal analgesia protocols. Moreover, extended follow‐ups are essential to determine whether the observed trends in pain relief translate into lasting outcomes.

## 5. Conclusion

In conclusion, within the context of a multimodal analgesia protocol for TKA under spinal anesthesia, a single low‐dose intraoperative IV ketamine bolus was not associated with a statistically or clinically meaningful reduction in postoperative opioid consumption or pain scores. However, given the limited power to detect small between‐group differences, these findings do not exclude a modest opioid‐sparing effect. Further research is essential to explore alternative dosing and routes, various analgesia protocols, and follow‐up durations when utilizing ketamine for pain management.

## Author Contributions

This study was designed by Arash Sherafat Vaziri, Seyedeh Reihaneh Hosseini, and Sina Javidmehr. The experiments were performed by Arash Sherafat Vaziri, Alireza Arvin, Seyedeh Reihaneh Hosseini, Fardis Vosoughi, Hassan Zolghadr, and Sohrab Keyhani. The data were analyzed by Mir Saeed Yekaninejad and Maryam Salimi, and the results were critically examined by all authors. Arash Sherafat Vaziri and Arash Heroabadi had a primary role in preparing the manuscript, which was edited by Alireza Arvin, Fardis Vosoughi, and Sohrab Keyhani.

## Funding

The authors have nothing to report.

## Disclosure

All authors have approved the final version of the manuscript and agree to be accountable for all aspects of the work.

## Conflicts of Interest

The authors declare no conflicts of interest.

## Supporting Information

Additional supporting information can be found online in the Supporting Information section.

## Supporting information


**Supporting Information 1** Supporting Appendix S1 provides the opioid oral morphine milligram equivalent (MME) conversion factors used to calculate daily postoperative opioid consumption. The appendix was used to standardize opioid doses into MME/day according to CDC analytic guidelines.

**Supporting Information 2** CONSORT 2010 checklist of information to include when reporting a randomised trial.

## Data Availability

The data that support the findings of this study are available from the corresponding author upon reasonable request.
