# Supplementary material for: Efficacy of Low‐Dose Intravenous Ketamine With Spinal Anesthesia for Postoperative Pain Control in Total Knee Arthroplasty: A Randomized Clinical Trial
Source: Pain Res Manag. 2026 Jun 17;2026:8176907. doi: 10.1155/prm/8176907 (PMC13273836; doi:10.1155/prm/8176907)
Supplement: Supplementary file 1 — Supporting Information 1 Supporting Appendix S1 provides the opioid oral morphine milligram equivalent (MME) conversion factors used to calculate daily postoperative opioid consumption. The appendix was used to standardize opioid doses into MME/day according to CDC analytic guidelines. [file PRM-2026-8176907-s002.pdf]

Opioid Oral Morphine Milligram Equivalent (MME) Conversion Factors[i,ii]

**Type of Opioid (strength units)**	**MME Conversion Factor**
Buprenorphine film/tablet[iii] (mg)	
Buprenorphine patch[iii] (mcg/hr)	
Buprenorphine film [iii] (mcg)	
Butorphanol (mg)	7
Codeine (mg)	0.15
Dihydrocodeine (mg)	0.25
Fentanyl buccal or SL tablets, or lozenge/troche[iv] (mcg)	0.13
Fentanyl film or oral spray[v] (mcg)	0.18
Fentanyl nasal spray[vi] (mcg)	0.16
Fentanyl patch[vii] (mcg)	7.2
Hydrocodone (mg)	1
Hydromorphone (mg)	4
Levorphanol tartrate (mg)	11
Meperidine hydrochloride (mg)	0.1
Methadone[viii] (mg)	3
>0, <= 20	4
>20, <=40	8
>40, <=60	10
>60	12
Morphine (mg)	1
Opium (mg)	1
Oxycodone (mg)	1.5
Oxymorphone (mg)	3
Pentazocine (mg)	0.37
Tapentadol[ix] (mg)	0.4
Tramadol (mg)	0.1

---

[i] The MME conversion factor is intended only for analytic purposes where prescription data are used to calculate daily MME. Use the formula: Strength per Unit X (Number of Units/ Days Supply) X MME conversion factor = MME/Day. This value does not constitute clinical guidance or recommendations for converting patients from one form of opioid analgesic to another. Please consult the manufacturer's full prescribing information for such guidance. Use of this file for the purposes of any clinical decision-making warrants caution. This is particularly true with regard to methadone (see viii below).

[ii] National Center for Injury Prevention and Control. CDC compilation of benzodiazepines, muscle relaxants, stimulants, zolpidem, and opioid analgesics with oral morphine milligram equivalent conversion factors, 2017 version. Atlanta, GA: Centers for Disease Control and Prevention; Available at https://www.cdc.gov/drugoverdose/resources/data.html . For more information, send an email to Mbohm@cdc.gov.

[iii] Buprenorphine products are listed but do not have an associated MME conversion factor. These buprenorphine products, as partial opioid agonists, are not expected to be associated with overdose risk in the same dose-dependent manner as doses for

full agonist opioids. The conversion factors for drugs prescribed or provided as part of medication-assisted treatment for opioid use disorder should not be used to benchmark against dosage thresholds meant for opioids prescribed for pain.

[iv] The MME conversion factor for fentanyl buccal tablets, sublingual tablets, and lozenges/troche is 0.13. This conversion factor should be multiplied by the number of micrograms in a given tablet or lozenge/troche.

[v] The MME conversion factor for fentanyl film and oral spray is 0.18. This reflects a 40% greater bioavailability for films compared to lozenges/tablets and 38% greater bioavailability for oral sprays compared to lozenges/tablets.

[vi] The MME conversion factor for fentanyl nasal spray is 0.16, which reflects a 20% greater bioavailability for sprays compared to lozenges/tablets.

[vii] The MME conversion factor for fentanyl patches is based on the assumption that one milligram of parenteral fentanyl is equivalent to 100 milligrams of oral morphine and that one patch delivers the dispensed micrograms per hour over a 24 hour day.
Example: 25 ug/hr fentanyl patch X 24 hrs = 600 ug/day fentanyl = 60 mg/day oral morphine milligram equivalent.
In other words, the conversion factor not accounting for days of use would be 60/25 or 2.4.
However, since the fentanyl patch remains in place for 3 days, we have multiplied the conversion factor by 3 (2.4 X 3 = 7.2). In this example, MME/day for ten 25 µg/hr fentanyl patches dispensed for use over 30 days would work out as follows:
Example: 25 ug/hr fentanyl patch X (10 patches/30 days) X 7.2 = 60 MME/day. Please note that because this allowance has been made based on the typical dosage of one fentanyl patch per 3 days, you should first change all Days Supply in your prescription data to follow this standard, i.e., Days Supply for fentanyl patches= # of patches X 3.

[viii] The CDC MME conversion factor to calculate morphine milligram equivalents of methadone is 3. Calculating MME for methadone in clinical practice often involves a sliding-scale approach whereby the conversion factor increases with increasing dose since the conversion factor of 3 for methadone could underestimate MME for a given patient. CMS uses this conversion factor when analyzing Medicare population opioid use. CMS uses the graduated methadone MME conversion factors to calculate MME within the Overutilization Monitoring System (OMS) for identifying and reporting potential opioid overutilizers. https://www.cdc.gov/drugoverdose/pdf/calculating_total_daily_dose-a.pdf.

[ix] Tapentadol is a mu receptor agonist and norepinephrine reuptake inhibitor. Oral MMEs are based on degree of mu receptor agonist activity, but it is unknown if this drug is associated with overdose in the same dose-dependent manner as observed with medications that are solely mu receptor agonists.
